# A bispecific nanobody dimer broadly neutralizes SARS-CoV-1 & 2 variants of concern and offers substantial protection against Omicron via low-dose intranasal administration

**DOI:** 10.1038/s41421-022-00497-w

**Published:** 2022-12-09

**Authors:** Huan Ma, Xinghai Zhang, Weihong Zeng, Junhui Zhou, Xiangyang Chi, Shaohong Chen, Peiyi Zheng, Meihua Wang, Yan Wu, Dan Zhao, Fanwu Gong, Haofeng Lin, Hancong Sun, Changming Yu, Zhengli Shi, Xiaowen Hu, Huajun Zhang, Tengchuan Jin, Sandra Chiu

**Affiliations:** 1 Department of Pulmonary and Critical Care Medicine, The First Affiliated Hospital of USTC, Division of Life Sciences and Medicine, University of Science and Technology of China, Hefei, Anhui China; 2 State Key Laboratory of Virology, Wuhan Institute of Virology, Center for Biosafety Mega-Science, Chinese Academy of Sciences, Wuhan, Hubei China; 3 Division of Life Sciences and Medicine, University of Science and Technology of China, Hefei, Anhui China; 4 University of Chinese Academy of Sciences, Beijing, China; 5 Institute of Biotechnology, Academy of Military Medical Sciences, Beijing, China; 6 CAS Key Laboratory of Special Pathogens, Wuhan Institute of Virology, Chinese Academy of Sciences, Wuhan, Hubei China; 7 Institute of Health and Medicine, Hefei Comprehensive National Science Center, Hefei, Anhui China

**Keywords:** Immunology, X-ray crystallography

## Abstract

Current SARS-CoV-2 Omicron subvariants impose a heavy burden on global health systems by evading immunity from most developed neutralizing antibodies and vaccines. Here, we identified a nanobody (aSA3) that strongly cross-reacts with the receptor binding domain (RBD) of both SARS-CoV-1 and wild-type (WT) SARS-CoV-2. The dimeric construct of aSA3 (aSA3-Fc) tightly binds and potently neutralizes both SARS-CoV-1 and WT SARS-CoV-2. Based on X-ray crystallography, we engineered a bispecific nanobody dimer (2-3-Fc) by fusing aSA3-Fc to aRBD-2, a previously identified broad-spectrum nanobody targeting an RBD epitope distinct from aSA3. 2-3-Fc exhibits single-digit ng/mL neutralizing potency against all major variants of concerns including BA.5. In hamsters, a single systemic dose of 2-3-Fc at 10 mg/kg conferred substantial efficacy against Omicron infection. More importantly, even at three low doses of 0.5 mg/kg, 2-3-Fc prophylactically administered through the intranasal route drastically reduced viral RNA loads and completely eliminated infectious Omicron particles in the trachea and lungs. Finally, we discovered that 2(Y29G)-3-Fc containing a Y29G substitution in aRBD-2 showed better activity than 2-3-Fc in neutralizing BA.2.75, a recent Omicron subvariant that emerged in India. This study expands the arsenal against SARS-CoV-1, provides potential therapeutic and prophylactic candidates that fully cover major SARS-CoV-2 variants, and may offer a simple preventive approach against Omicron and its subvariants.

## Introduction

SARS-CoV-2 (hereafter, SARS2) has been spreading for more than two and a half years, causing huge losses to the global economy and human health. In addition to active immunization of vaccines, passive administration of neutralizing antibodies is a promising therapeutic against the virus infection^1,2^. However, the continued adaptive evolution of the virus leads to the emergence of variants of concern (VOCs), especially the currently circulating Omicron subvariants, which have evaded most available antibodies and reduced the effectiveness of vaccines designed on the basis of the original strain^3–10^. Prior to SARS2, the closely analogous coronavirus, SARS-CoV-1 (hereafter, SARS1), emerged in 2002–2003 and was associated with much higher mortality than SARS2^11^, but there are currently no approved treatments against it. Thus, ideal countermeasure candidates would be able to effectively treat and prevent both SARS1 and SARS2 and their emerging variants. Both SARS2 and SARS1 infect humans mainly through contact with cells expressing angiotensin-converting enzyme 2 (ACE2) in the respiratory tract, and respiratory viral load positively correlates with disease severity in infected individuals^12–16^, suggesting that direct delivery of antiviral antibodies to the respiratory tract would achieve the desired efficacy at low doses.

Multivalent engineering, including artificial homo- and hetero-multivalences is a successful approach that significantly enhances overall avidity as well as neutralization potency^17–20^. Multivalent antibodies are more cost-effective due to their simpler formulation and manufacture compared to antibody cocktails^21^. Nevertheless, the multivalent engineering of conventional antibodies is complicated by the fact that the variable regions of the heavy and light chains contribute jointly to antigen binding. Nanobodies (Nbs, also called VHH) consist of only one Ig domain and thus are easier to engineer into multivalent formats than conventional antibodies^22^. Moreover, Nbs are thermally resistant and stable against harsh conditions and extreme pH, and thus are superior to conventional antibodies in storage and transportation^23^, which are critical in response to emerging pandemics.

We previously developed a broad-spectrum SARS2 neutralizing Nb named aRBD-2, which targets a conserved epitope on the receptor binding motif (RBM) of the SARS2 RBD and thus broadly binds all VOCs^24^. In this study, we report a newly identified Nb termed aSA3, which targets an RBD core epitope distinct from aRBD-2 and cross-reacts with SARS1, wild-type (WT) SARS2, and all major VOCs. Based on aSA3 and aRBD-2, we engineered a novel bispecific Nb dimer termed 2-3-Fc, which robustly neutralizes all VOCs, including BA.5 and the recent BA.2.75. In vivo assays showed that 2-3-Fc was effective in combating Omicron infection through not only a normal dose of intraperitoneal (i.p.) injection but also a low dosage of intranasal (i.n.) administration.

## Results

### aSA3 cross-reacts with SARS1 and WT SARS2 by targeting an epitope on the RBD core

**Fig. 1:**
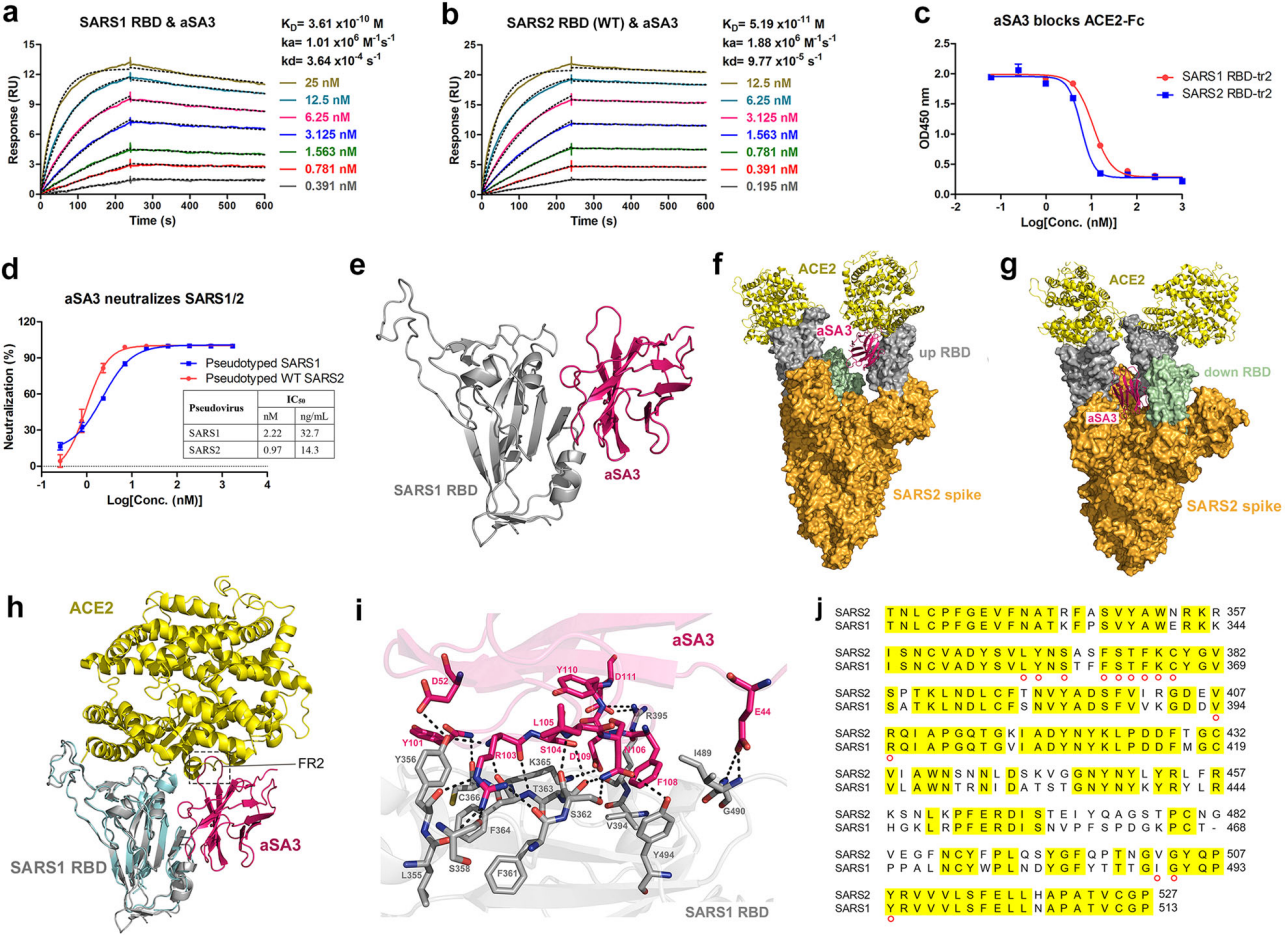
aSA3 cross-reacts with SARS1 and WT SARS2 by targeting an epitope on RBD core. **a**, **b** The binding kinetics of aSA3 to SARS1 RBD (**a**) and WT SARS2 RBD (**b**) were monitored by the Biacore 8K system. The actual responses (colored lines) and the data fitted to a 1:1 binding model (black dotted lines) are shown. *K*_D_, equilibrium dissociation constant; *k*_a_, association constant; *k*_d_, dissociation constant. **c** The blocking abilities of aSA3 against the interaction between ACE2-Fc and SARS1/2 RBD were tested by competitive ELISA. SARS1 RBD-tr2 or WT SARS2 RBD-tr2 were coated on the plate and incubated with a mixture of 20 nM ACE2-Fc with serial dilutions of aSA3. Bound ACE2-Fc was detected with HRP conjugated anti-IgG1 Fc antibody. Error bars indicate the means ± SD from two independent experiments. **d** The neutralizing activities of aSA3 against pseudotyped SARS1 and WT SARS2. The IC_50_ values of the pseudovirus neutralization assay were calculated by fitting the inhibition rates against antibody concentrations with a sigmoidal dose-response curve. Error bars indicate the means ± SD from triplicates. **e** The overall structure of aSA3 (hotpink) bound to SARS1 RBD (gray) using cartoon presentation. **f**, **g** Superposition analysis of aSA3 onto one “up” RBD (**f**) and one “down” RBD (**g**) in the cryo-EM structure of the trimeric spike of SARS2 (PDB: 7KMZ). **h** Structure alignment of the aSA3:SARS1 RBD complex with the ACE2:SARS1 RBD complex (PDB: 2AJF). The dotted box marks the region where FR2 of aSA3 clashes with ACE2. **i** Zoom-in view of the interaction interface of aSA3 and SARS1 RBD. Interacting residues are shown as sticks, and dotted lines indicate hydrogen bonds and salt bridges. **j** Sequence alignment of the SARS1 and SARS2 RBDs. Identical residues are marked with yellow shading, and residues interacting with aSA3 are indicated with red circles.

We previously immunized two alpacas with the RBD protein of WT Wuhan isolated SARS2 and constructed a phage library displaying VHH derived from peripheral blood mononuclear cells^25^. The library was panned against the SARS1 RBD for two rounds and yielded a panel of Nbs cross-reactive with the RBD of SARS1 and WT SARS2 (data not shown). Among them, a Nb named aSA3 tightly bound to the RBD of SARS1 and WT SARS2 with equilibrium dissociation constant (*K*_D_) values of 361 pM and 51.9 pM, respectively (Fig. 1a, b), was selected for further in-depth characterization. Competitive enzyme-linked immunosorbent assay (ELISA) revealed that aSA3 dose-dependently blocked the interaction between hACE2-Fc (extracellular domain of human ACE2 fused with human IgG1 Fc) and the RBD of SARS1 or WT SARS2 (Fig. 1c). Benefiting from the high-affinity RBD binding and effective ACE2 blockage, aSA3 potently neutralized SARS1 (IC_50_: 2.22 nM or 32.7 ng/mL) and WT SARS2 (IC_50_: 0.97 nM or 14.3 ng/mL) in pseudovirus neutralization assay (Fig. 1d).

To gain insight into the mechanism by which aSA3 strongly cross-reacts with SARS1 and WT SARS2, we determined the crystal structure of aSA3 in complex with SARS1 RBD-tr2 (RBD tandem repeat dimer) at a resolution of 3.38 Å by molecular replacement [Protein Data Bank (PDB) ID: 7X4I] (Fig. 1e). Data collection and model refinement statistics of the structure are shown in Supplementary Table S1. The electron density signature is well defined and covers most interacting residues of SARS1 RBD and aSA3. Superposition of the structure onto the RBD of the WT SARS2 spike conformations resolved by cryo-electron microscopy (cryo-EM)^26^ indicates that aSA3 binds to an RBD core region on the inner side of the RBD, which is buried when the spike protein is in a closed state (Fig. 1f). Due to steric hindrance with adjacent RBDs, aSA3 can only bind to the “up” RBD but not to the “down” RBD (Fig. 1g). Although the footprint of aSA3 on the RBD does not overlap with that of ACE2, the extended FR2 region of aSA3 would clash with ACE2 when in complex with SARS1 RBD (Fig. 1h), explaining the ACE2–RBD blockage of aSA3.

According to the structure, FR2, CDR2, and CDR3 of aSA3 are involved in binding SARS1 RBD and bury a surface area of 877.8 Å^2^. Overall, fourteen residues of SARS1 RBD, including L355, Y356, S358, F361, S362, T363, F364, K365, C366, V394, R395, I489, G490, and Y494, are closely contacted by aSA3 (Fig. 1i). The detailed interactions between aSA3 and SARS1 RBD are shown in Supplementary Table S2. According to the amino acid sequence alignment, except for I489, all of the rest thirteen aSA3 contacting residues on SARS1 RBD are also present on SARS2 RBD (Fig. 1j). Since I489 of SARS1 RBD binds aSA3 through its main chain amide group, the counterpart residue V503 of SARS2 RBD should also bind aSA3. These analyses explain the tight binding and neutralization efficacy of aSA3 to both SARS1 and SARS2. The counterpart SARS2 RBD residues closely bound by aSA3 are L368, Y369, S371, F374, S375, T376, F377, K378, C379, V407, R408, V503, G504, and Y508 according to the sequence alignment (Fig. 1j).

### aSA3-Fc potently neutralizes SARS1, WT SARS2 and VOCs except for BA.2 and BA.5

**Fig. 2:**
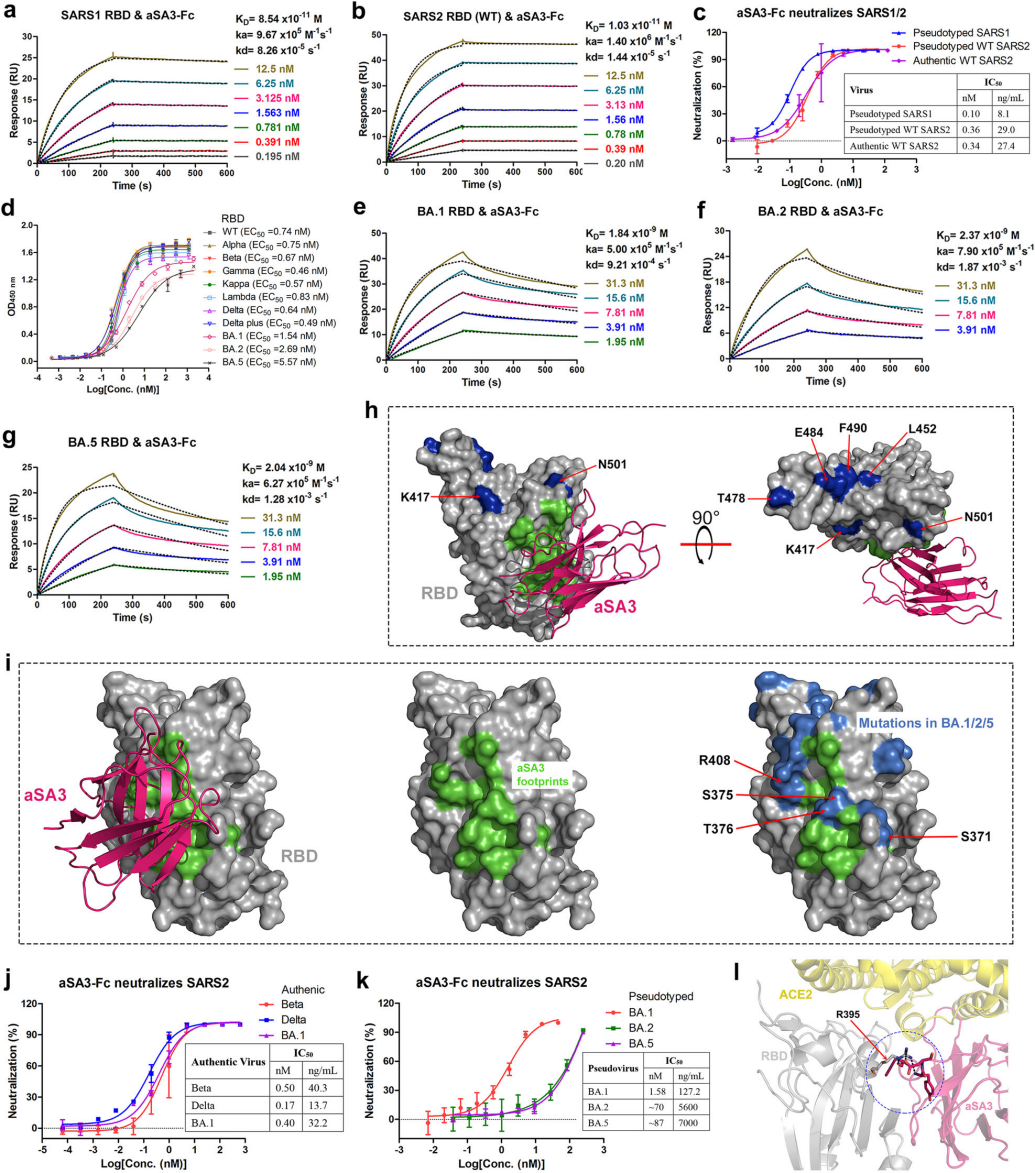
aSA3-Fc is affected by mutations in the Omicron RBD. **a**, **b** SPR binding kinetics of aSA3-Fc to SARS1 RBD (**a**) and WT SARS2 RBD (**b**) were monitored by the Biacore 8 K system. The actual responses (colored lines) and the data fitted to a 1:1 binding model (black dotted lines) are shown. **c** The neutralizing activities of aSA3-Fc against SARS1 and WT SARS2. Pseudovirus neutralization assays were performed to characterize the neutralizing activities of aSA3-Fc against SARS1 and WT SARS2 pseudoviruses, while PRNT was conducted to assess the neutralizing activities of aSA3-Fc against authentic WT SARS2. **d** ELISA results for the binding of aSA3-Fc to the RBD of WT SARS2 and its major variants. EC_50_ values were calculated by fitting the OD_450_ values to a sigmoidal dose-response curve and shown in the brackets. **e**–**g** SPR binding kinetics of aSA3-Fc to the RBD of Omicron BA.1 (**e**), BA.2 (**f**), and BA.5 (**g**). The actual responses (colored lines) and the data fitted to a 1:1 binding model (black dotted lines) are shown. **h** The structure of aSA3:SARS1 RBD complex superimposed on the structure of SARS2 RBD (PDB: 6M0J). The six mutation sites shared by the RBDs of Alpha, Beta, Gamma, Kappa, Lambda, Delta, and Delta plus are marked in blue, the aSA3 footprints are marked in green. **i** The aSA3 footprints (green) and mutation sites of BA.1, BA.2, and BA.5 (marine) on RBD. **j** The neutralization properties of aSA3-Fc against authentic Beta, Delta and BA.1. **k** The neutralization properties of aSA3-Fc against pseudotyped BA.1, BA.2, and BA.5. The IC_50_ values were calculated by fitting the inhibition rates against antibody concentrations with a sigmoidal dose-response curve. Error bars indicate the means ± SD from three (pseudovirus) or two replicates (authentic virus). **l** R408S mutation present on BA.2 and BA.5 (R408 of SARS2 RBD corresponds to R395 of SARS1 RBD) may reduce the binding of aSA3 to the α-helix on the concave surface of RBD. Black dotted lines indicate hydrogen bonds and salt bridges.

We then constructed aSA3-Fc by fusing aSA3 to human IgG1 Fc as described in our previous study^25^, which demonstrated that the Nb-Fc homodimer showed improved binding and neutralizing activity compared to the Nb monomer. As expected, aSA3-Fc bound the SARS1 RBD (*K*_D_: 85.4 pM) and WT SARS2 RBD (*K*_D_: 10.3 pM) with 4- and 5-fold higher affinity (Fig. 2a, b) and neutralized pseudotyped SARS1 (IC_50_: 0.10 nM or 8.1 ng/mL) and pseudotyped WT SARS2 (IC_50_: 0.36 nM or 29.0 ng/mL) with 22- and 3-fold more potency (on a molar basis) than aSA3 did, respectively (Fig. 2c). Consistently, aSA3-Fc also potently neutralized authentic WIV1 (a bat coronavirus closely related to SARS1^27^) (observed IC_50_: 1.04 nM or 83.7 ng/mL) in micro-neutralization test and authentic WT SARS2 (IC_50_: 0.34 nM or 27.4 ng/mL) in plaque reduction neutralization test (PRNT; Fig. 2c).

Since emerging VOCs have escaped most developed antibodies, we then investigated the binding and neutralizing properties of aSA3-Fc to the major VOCs. ELISA revealed that aSA3-Fc retained tight binding to the RBDs of Alpha, Beta, Gamma, Kappa, Lambda, Delta, and Delta plus but had decreased binding to the RBDs of Omicron BA.1, BA.2, and BA.5 (Fig. 2d). Further Surface plasmon resonance (SPR) assays showed that aSA3-Fc bound the three Omicron RBDs with *K*_D_ values ranging from 2.37 nM to 1.84 nM, approximately 200 times higher than its *K*_D_ value for the WT RBD (Fig. 2e–g). Structurally, aSA3 does not form extensive interactions with K417, L452, T478, E484, F490, and N501, which are mutated in the RBDs of Alpha, Beta, Gamma, Kappa, Lambda, Delta and Delta plus variants (Fig. 2h), explaining the resistance of aSA3 to these variants. By contrast, the mutations at S371 and S375 (corresponding to S358 and S362 of SARS1 RBD) in Omicron BA.1 and additional mutations at T376 and R408 (corresponding to T363 and R395 of SARS1 RBD) in both BA.2 and BA.5 overlap with aSA3 footprints, which should be the reason for the greatly deceased affinities of aSA3 to them (Fig. 2i).

We next tested the potency of aSA3-Fc in neutralizing major SARS2 variants. PRNT showed that aSA3-Fc potently neutralized Beta (IC_50_: 0.50 nM or 40.3 ng/mL) and Delta (IC_50_: 0.17 nM or 13.7 ng/mL) in consistent with the binding data, and surprisingly, it also potently neutralized BA.1 (IC_50_: 0.40 nM or 32.2 ng/mL), despite the greatly reduced binding affinity (Fig. 2j). Nevertheless, pseudovirus neutralization assays showed that aSA3-Fc only potently neutralized BA.1 (IC_50_: 1.58 nM or 127.2 ng/mL) but weakly neutralized BA.2 (IC_50_: ~70 nM or 5.6 μg/mL) and BA.5 (IC_50_: ~87 nM or 7.0 μg/mL) (Fig. 2k). The neutralizing activity against BA.2 and BA.5 was reduced by over 40 times compared to that against BA.1. According to structural information, the R408S mutation present on BA.2 and BA.5 but not on BA.1 could cause aSA3 to lose its anchor to the α-helix on the concave surface of the RBD (Fig. 2l), which possibly renders aSA3 less effective in blocking the ACE2–RBD interaction and thus less potent in neutralizing BA.2 and BA.5, explaining the different performance of aSA3-Fc in neutralizing BA.1 versus BA.2 and BA.5.

### Bispecific antibody 2-3-Fc designed by fusing aSA3-Fc to nonoverlapping aRBD-2 potently neutralizes all VOCs

**Fig. 3:**
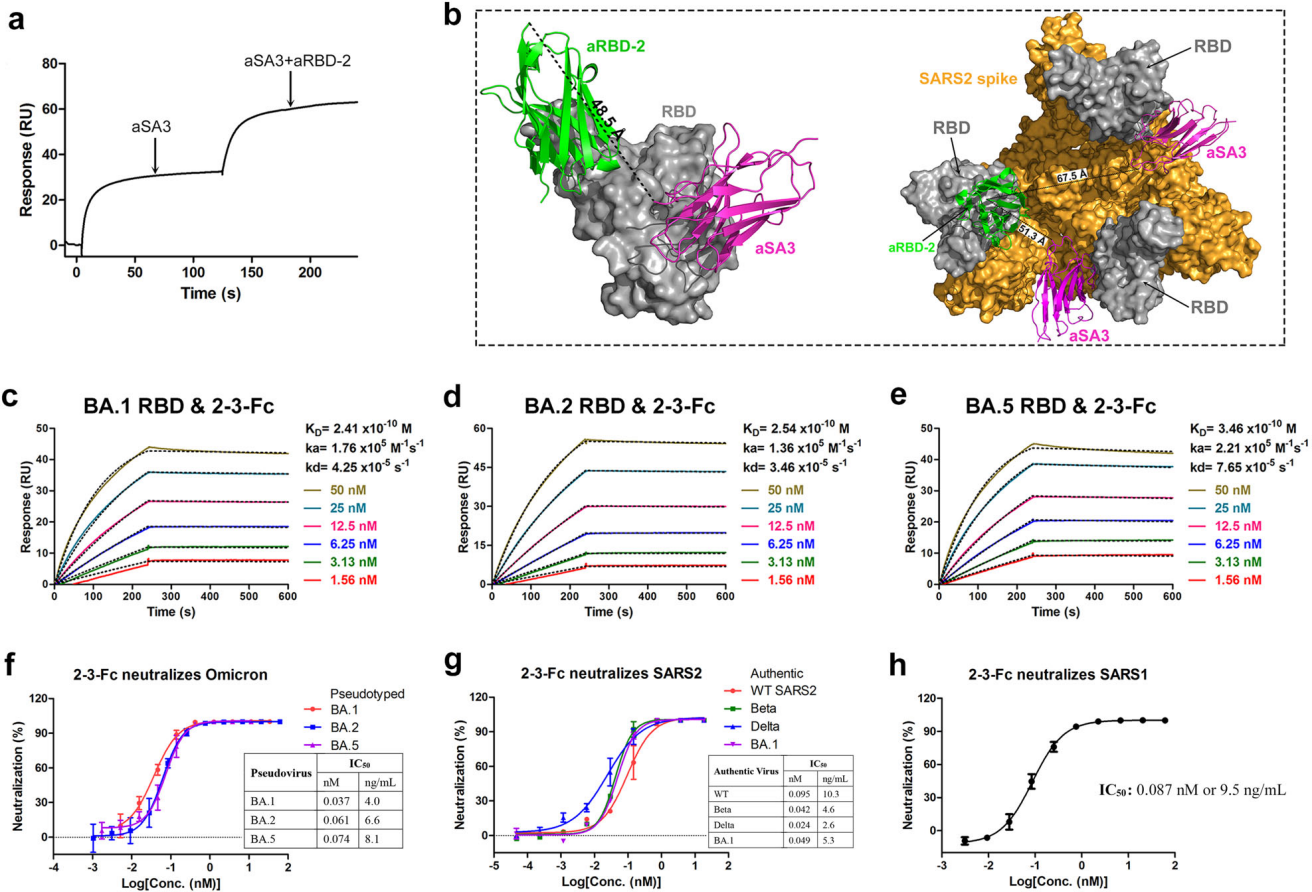
2-3-Fc tightly binds and potently neutralizes all major VOCs. **a** Competition between aSA3 and aRBD-2 for binding to the SARS2 RBD was identified using SPR. SARS2 RBD was immobilized on CM5 chips, and aSA3 was injected for 120 s, followed by injection of a 1:1 mixture of aSA3 in combination with aRBD-2 for 120 s. **b** The structure of aRBD-2:SARS2 RBD complex (PDB ID: 7FH0) was aligned with that of the aSA3:SARS1 RBD complex (PDB ID: 7X4I) (left). The straight line distance between the C-terminus of aRBD-2 and the N-terminus of aSA3 on one RBD was measured as 48.5 Å. The structures of aRBD-2:SARS2 RBD complex and aSA3:SARS1 RBD complex were superimposed on the RBD in the cryo-EM structures of the trimeric spike with all RBDs in the “up” conformation (PDB: 7KMS) (right), and the distance between the C-terminus of aRBD-2 and the N-terminus of aSA3 on two adjacent RBDs was measured as 51.3 Å or 67.5 Å. The length of the flexible 4(G_4_S) linker is approximately 72 Å in an extended form. **c**–**e** SPR kinetics of 2-3-Fc to the RBD of Omicron BA.1 (**c**), BA.2 (**d**), and BA.5 (**e**). The actual responses (colored lines) and the data fitted to a 1:1 binding model (black dotted lines) are shown. **f** The neutralization properties of 2-3-Fc against pseudotyped BA.1, BA.2, and BA.5. **g** The neutralization properties of 2-3-Fc against authentic WT, Beta, Delta and BA.1 viruses. **h** The neutralization properties of 2-3-Fc against pseudotyped SARS1. The IC_50_ values were calculated by fitting the inhibition rates against antibody concentrations with a sigmoidal dose-response curve. Error bars indicate the means ± SD from three (pseudovirus) or two replicates (authentic virus).

We previously developed a broad-spectrum Nb, called aRBD-2, targeting a unique and highly conserved RBM epitope. We also demonstrated that the bispecific Nbs constructed by fusing two nonoverlapping RBM-targeting Nbs have highly improved performance than the parental Nbs in binding and neutralizing SARS2 variants, even if one of the two-component Nbs loses apparent binding to the variants^24^. Consistent with the structural information that aSA3 and aRBD-2 bind nonoverlapping epitopes, SPR assay showed that aSA3 and aRBD-2 can bind RBD simultaneously (Fig. 3a). Given that aSA3-Fc has a broad binding spectrum to major VOCs (albeit with reduced affinity for Omicron variants), we fused aSA3-Fc to the C-terminus of aRBD-2 to construct a novel bispecific Nb dimer, termed 2-3-Fc, which would be more resistant to emerging variants than the hetero-bivalent Nbs (aRBD-2-5-Fc and aRBD-2-7-Fc) we previously constructed^24^ due to its two active components. An optimized flexible Gly-Ser linker of 20 amino acids was inserted between aRBD-2 and aSA3-Fc, which allows aSA3 and aRBD-2 in 2-3-Fc to simultaneously bind the same RBD or two adjacent RBDs of the spike (Fig. 3b). As expected, 2-3-Fc exhibited higher binding affinities than aSA3-Fc and aRBD-2-Fc^24^, with *K*_D_ values in sub-nanomolar for the RBDs of Omicron BA.1, BA.2, and BA.5 (Fig. 3c–e), indicating synergistic binding of the two component Nbs. Consistent with the increased affinities, 2-3-Fc exhibited 40-, 1000-, and 1000-fold enhanced activity (on a molar basis) than aSA3-Fc in neutralizing pseudotyped Omicron BA.1, BA.2, and BA.5, with IC_50_ values of 0.037 nM (4.0 ng/mL), 0.061 nM (6.6 ng/mL), and 0.074 nM (8.1 ng/mL), respectively (Fig. 3f). 2-3-Fc also displayed further gains in potency in neutralizing authentic WT, Beta, Delta, and BA.1, with IC_50_ values of 0.095 nM (10.3 ng/mL), 0.042 nM (4.6 ng/mL), 0.024 nM (2.6 ng/mL), and 0.049 nM (5.3 ng/mL), respectively (Fig. 3g). Since aRBD-2 cannot bind SARS1 RBD, 2-3-Fc is not superior but comparable to aSA3-Fc in neutralizing SARS1, with an IC_50_ of 0.087 nM (9.5 ng/mL; Fig. 3h).

### 2-3-Fc protected hamsters against Omicron BA.1 infection via i.p. administration

**Fig. 4:**
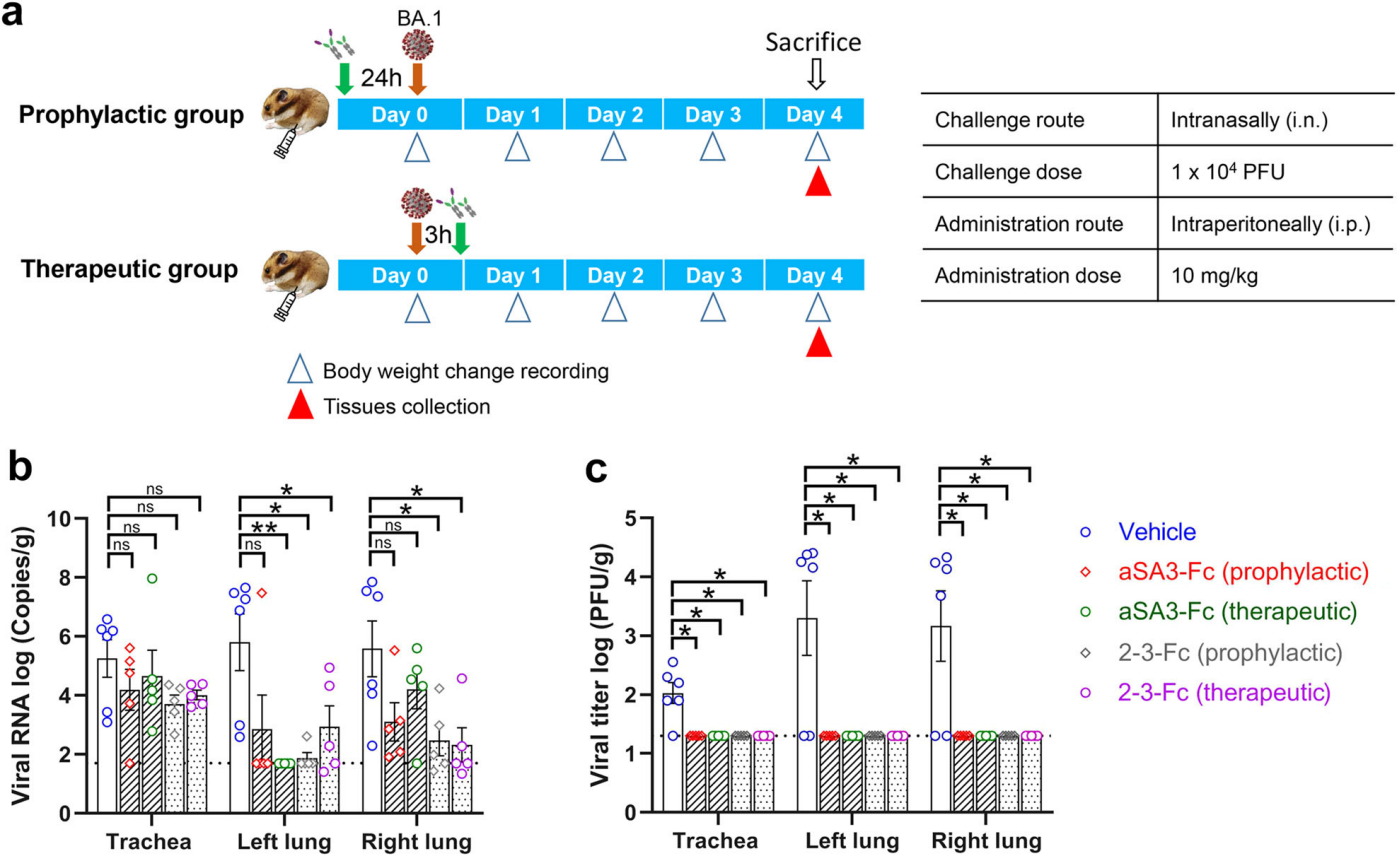
aSA3-Fc and 2-3-Fc provide prophylactic and therapeutic protection against Omicron BA.1 in hamsters via i.p. administration. **a** Animal experiment scheme. **b**, **c** Syrian hamsters were divided into five groups that were treated with vehicle (PBS) (*n* = 6), aSA3-Fc (*n* = 5) and 2-3-Fc (*n* = 5) before or after the i.n. challenge with Omicron BA.1 virus. At 4 dpi, viral RNA copies (**b**) and viral titers of infectious virus (**c**) in the trachea and lungs of hamsters were measured with qPCR and plaque assays, respectively. Error bars indicate the means ± SEM. The animal experiment was performed in parallel and shared the vehicle control group with our another study^24^.

The observed very potent neutralizing activity of 2-3-Fc against Omicron variants prompted us to test its in vivo protection efficacy. Since the authorized SARS2 neutralizing antibodies for emergency use are all administered by systemic infusion, we first assessed the in vivo efficacy of 2-3-Fc against Omicron BA.1 through i.p. injection using a previously described hamster model^28^. aSA3-Fc was tested in parallel as it also effectively neutralized BA.1. 10 mg per kg body weight (hereafter, mg/kg) of the antibodies were i.p. administered to animals before (prophylactic group) or after (therapeutic group) i.n. challenge with authentic BA.1 virus. The hamsters treated with PBS (vehicle) were set as a control group (Fig. 4a). Viral RNA copies (Fig. 4b) and titers of infectious virus (Fig. 4c) in the trachea and lungs of the animals were determined at 4 days post-infection (dpi). Overall, the mean RNA copies in the animals treated prophylactically or therapeutically were reduced as compared to those in the control group (Fig. 4b). Specifically, for aSA3-Fc treatment, the mean RNA copies in the trachea, left lung and right lung of the animals in the prophylactic group were reduced by 10^1.07^-, 10^2.96^-, and 10^2.48^-fold, respectively, while those in the therapeutic group were reduced by 10^0.60^-, 10^4.11^-, and 10^1.38^-fold, respectively. In the tissues of 2-3-Fc treated animals, the mean viral RNA decreased by 10^1.55^-, 10^3.93^-, and 10^3.11^-fold for the prophylactic group and 10^1.25^-, 10^2.88^-, and 10^3.26^-fold for the therapeutic group, respectively. Importantly, infectious virus was completely abrogated in the tissues of all the treated animals. In contrast, a significant amount of infectious virus was still detected in the control group (Fig. 4c). Animal body weight loss and mortality were not observed (data not shown), as Omicron BA.1 caused only attenuated disease in hamsters^29^. These results demonstrate that both aSA3-Fc and 2-3-Fc administered at a single dose of 10 mg/kg through i.p. injection can offer effective in vivo prophylactic and therapeutic protection against Omicron BA.1.

### 2-3-Fc protected hamsters against Omicron infection through nasal delivery

**Fig. 5:**
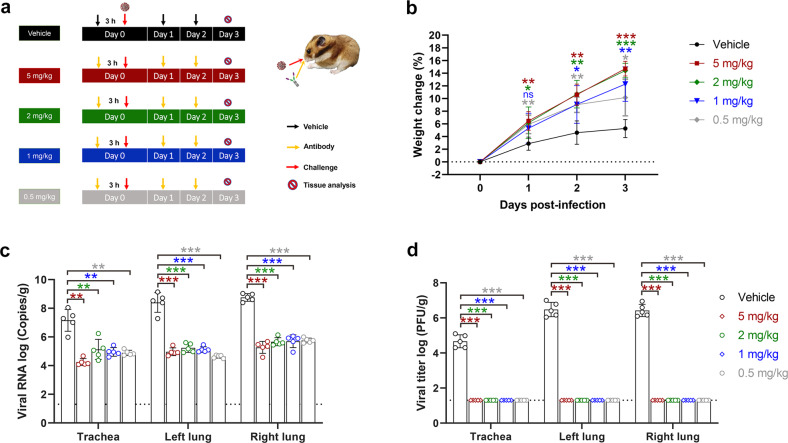
I.n. delivered 2-3-Fc offers prevention against Omicron in hamsters. **a** Animal experiment scheme. Hamsters were divided into 5 groups (*n* = 5 per group), including a control group administered vehicle (PBS) and four treatment groups administered doses of 5 mg/kg, 2 mg/kg, 1 mg/kg, and 0.5 mg/kg. **b** The body weight change of the animals in the control group and treatment groups was recorded daily and compared. **c**, **d** Viral RNA copies (**c**) and viral titers of infectious particles (**d**) in the trachea and lungs were measured at 3 dpi. Bars indicate the means ± SEM.

Respiratory tract is the main target of SARS2^14,30^, so the direct delivery of antiviral drugs to the respiratory system is expected to improve drug efficacy. I.n. delivery is a non-invasive and simple method of administration that can be widely used in the population to prevent SARS2 infection, so we further evaluated the prophylactic efficacy of 2-3-Fc against Omicron BA.1 using i.n. administration in hamsters. Five groups of animals were treated with three doses of 5 mg/kg, 2 mg/kg, 1 mg/kg, and 0.5 mg/kg of 2-3-Fc or PBS with the first dose at 3 h before the challenge, plus two more doses at 24 h and 48 h after the challenge **(**Fig. 5a**)**. Body weight changes were monitored daily. Animals were euthanized at 3 dpi, and viral RNA copies and titers of infectious particles in the trachea and lungs were determined. Although weight loss was not observed in the control group, the antibody-treated animals gained significantly more weight than the control animals, and a dose-dependent effect was observed in the 5 mg/kg and 2 mg/kg groups compared with the 0.5 mg/kg group (Fig. 5b). Average viral RNA copies in the trachea and lungs were significantly reduced in all four treatment groups compared to the control group. Specifically, the mean viral RNA copies in the trachea, left lung and right lung of the animals in the four treated groups were reduced by 10^2.03^- to 10^2.91^-fold, 10^3.17^- to 10^3.78^-fold, and 10^3.00^- to 10^3.48^-fold, respectively. No dose-dependent viral RNA reduction was observed in the tissues (Fig. 5c). Infectious viruses in the trachea and lungs were completely abrogated for all four treatment groups, while averages of 10^4.67^, 10^6.48^, and 10^6.43^ PFU/g infectious viruses were still present in the trachea, left lung and right lung of the control animals (Fig. 5d). These results demonstrate that nasal administration of 2-3-Fc can offer prophylactic protection against Omicron BA.1 in vivo even at the dose as low as 0.5 mg/kg.

### 2-3-Fc with Y29G substitution in aRBD-2 potently neutralizes Omicron BA.2.75

**Fig. 6:**
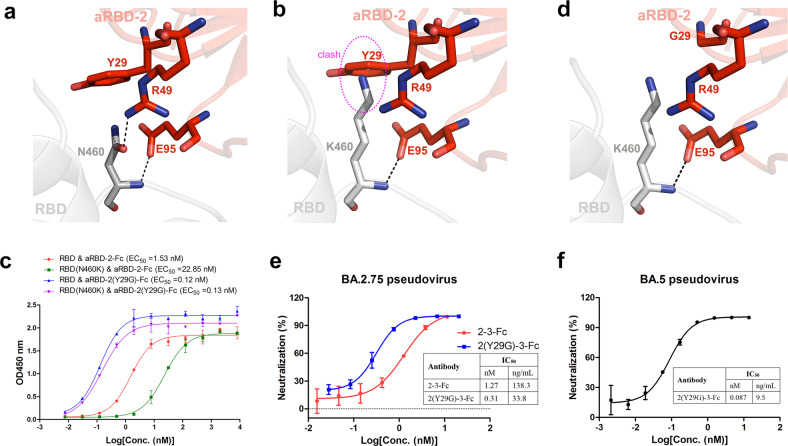
2(Y29G)-3-Fc is more potent than 2-3-Fc in neutralizing Omicron BA.2.75. **a**, **b** Zoom-in views of the structure of aRBD-2:SARS2 RBD complex (PDB ID: 7FH0) shows that N460 of SARS2 RBD is located at the edge of the binding interface and overlaps with the footprint of aRBD-2 (**a**), and the N460K mutation would cause the loss of a hydrogen bond interaction between N460 of RBD and R49 of aRBD-2 and create a steric clash with the side chain of Y29 of aRBD-2 (**b**). **c** ELISA results for the binding activities of aRBD-2-Fc and aRBD-2(Y29G)-Fc to the WT SARS2 RBD or WT SARS2 RBD with N460K mutation. EC_50_ values were calculated by fitting the OD_450_ values to a sigmoidal dose-response curve and are shown in the brackets. **d** A magnified view of the hypothetical structure shows that K460 of the RBD does not clash with G29 of aRBD-2. **e** The neutralization properties of 2-3-Fc and 2(Y29G)-3-Fc against pseudotyped BA.2.75. **f** The neutralization properties of 2(Y29G)-3-Fc against pseudotyped BA.5. The IC_50_ values were calculated by fitting the inhibition rates against antibody concentrations with a sigmoidal dose-response curve. Error bars indicate the means ± SD from triplicates.

Recently, another Omicron subvariant, BA.2.75, emerged in India and has been detected in at least 15 countries^31^. Compared with other Omicron subvariants, BA.2.75 carries an additional N460K mutation, which is located at the edge of the binding interface and within the footprint of aRBD-2 (Fig. 6a). The mutation would cause the loss of a hydrogen bond interaction between N460 of RBD and R49 of aRBD-2, and the long side chain of K460 formed by the mutation would create steric hindrance with the side chain of Y29 of aRBD-2 (Fig. 6b), and thus may weaken the binding of aRBD-2. ELISA confirmed the speculation that aRBD-2 showed a 15-fold higher EC_50_ value to the RBD with N460K mutation than to the WT RBD (Fig. 6c). To strengthen the binding of aRBD-2 to the RBD with N460K mutation, we mutated Y29 of aRBD-2 to residues with a short side chain (Fig. 6d). We found that aRBD-2-Fc with Y29G mutation, termed aRBD-2(Y29G)-Fc, tightly bound to both the WT RBD and RBD with N460K mutation, and interestingly, aRBD-2(Y29G)-Fc was even more active than original aRBD-2-Fc in binding the WT RBD (Fig. 6c). Based on these findings, 2-3-Fc with Y29G mutation in aRBD-2, termed 2(Y29G)-3-Fc, was prepared. We did not observe a yield reduction of 2(Y29G)-3-Fc compared to 2-3-Fc. We next tested 2-3-Fc and 2(Y29G)-3-Fc in neutralizing BA.2.75 pseudovirus. In alignment with the reduced binding activity of aRBD-2 to the RBD with N460K mutation, 2-3-Fc showed 17- to 19-fold reduced activity against BA.2.75 than against BA.2 and BA.5, but remained at a moderate level, with an IC_50_ of 1.27 nM (138.3 ng/mL) (Fig. 6e). In congruence with the recovered binding of aRBD-2(Y29G) to the RBD with N460K mutation, 2(Y29G)-3-Fc had a 4-fold increased neutralizing potency against BA.2.75 compared to 2-3-Fc, with an IC_50_ of 0.31 nM (or 33.8 ng/mL) (Fig. 6e). Consistent with that Y29 of aRBD-2 is not involved in binding to RBD, 2(Y29G)-3-Fc is as effective as 2-3-Fc in neutralizing BA5, with an IC_50_ of 0.087 nM (or 9.5 ng/mL) (Fig. 6f). These findings ensure full coverage of the emerged Omicron subvariants of our engineered Nbs.

## Discussion

Both SARS1 and SARS2 utilize their RBD of the spike protein to engage human ACE2 on host cells for infection, making RBD a primary target for neutralizing antibody development. As a matter of fact, RBD-targeting antibodies represent one of the most effective therapeutics^32–35^. Although several high-breadth antibodies targeting cryptic epitopes on S2 subunit of spike have been reported, these antibodies are generally modest in neutralizing potency^36–39^. Therefore, the development of RBD-targeting antibodies with potent and broad-spectrum neutralizing activity against the emerging variants to contain the current pandemic is still necessary.

We aim to produce robust antibodies capable of cross-inhibiting SARS1 and SARS2. The identity between the SARS1 RBD (T320–P513) and SARS2 RBD (T333–P527) is 73.85%, and the different residues between them are mainly located on the RBM toward the ACE2 binding interface^40–42^. Therefore, it is a reasonable strategy to use the SARS1 RBD to screen the VHH library constructed from alpacas immunized with the SARS2 RBD to obtain Nbs targeting the relatively conserved RBD core. Using this strategy, aSA3, which tightly binds and potently neutralizes both SARS1 and SARS2 was identified (Fig. 1). According to the structure, the RBD residues in contact with aSA3 are also present on the closely related bat coronavirus WIV1^27^, explaining the neutralization of WIV1 by aSA3-Fc. aSA3 has a 7-fold higher binding affinity to SARS2 RBD than to SARS1 RBD (Fig. 1a, b), and the mechanism for this needs further study on the structure of the aSA3:SARS2 RBD complex. Prior studies^43,44^ demonstrated that SARS2 spike (or its RBD) binds hACE2 with higher affinity than SARS1 spike (or its RBD) does, which may be the reason that aSA3-Fc showed higher affinity for SARS2 RBD than for SARS1 RBD but opposite neutralizing performance against the two viruses in pseudovirus neutralization assay (Fig. 2a–c).

There reported two other Nbs, VHH-72^45^ and Fu2^46^, both of which target RBD epitopes similar to aSA3 and neutralize both SARS1 and SARS2. VHH72 closely contacts seven SARS1 RBD residues, including Y356, S358, F364, K365, C366, R426, and Y494, and binds SARS1 and SARS2 RBD with *K*_D_ values of 1.15 nM and 38.6 nM, respectively. Its Fc fusion (VHH72-Fc) neutralized SARS1 and WT SARS2 pseudoviruses with IC_50_ values higher than 2 μg/mL. Compared with VHH72-Fc, aSA3-Fc binds more residues shared by SARS1 and SARS2 (Fig. 1i) and has much higher binding (Fig. 2a, b) and neutralizing activities to both viruses (Fig. 2c). Fu2 interacts with F374, S375, F377, K378, C379, G381, and D405 of the SARS2 RBD in the major interface according to structural analysis and binds the SARS2 RBD with a *K*_D_ of 118 pM. Its Fc fusion showed potent neutralization against WT SARS2 pseudovirus (IC_50_ of 61 ng/mL) but significantly reduced neutralization against SARS1 pseudovirus (IC_50_ of 570 ng/mL). Compared with Fu2-Fc, aSA3-Fc has comparable neutralizing activity against SARS2 but much higher neutralizing activity against SARS1 (Fig. 2c).

We fused aSA3-Fc to aRBD-2 to construct a novel bispecific Nb dimer, 2-3-Fc, in which both components are active in binding to all VOCs. Neutralizing assays showed that 2-3-Fc very potently (nanogram per milliliter potency) neutralized all major VOCs, including Beta, Delta, Omicron BA.1, BA.2, and BA.5 (Fig. 3f, g), better than previously reported bispecific SARS2 neutralizing Nbs^47,48^. 2-3-Fc is expected be equally potent against BA.3, BA.1.1, BA.2.12.1, BA.2.9.1, BA.2.11, BA.4.6, and BJ.1 variants that were once or being under monitoring by WHO (https://www.who.int/activities/tracking-SARS-CoV-2-variants, as of 23 October 2022), as these variants do not have additional mutations in the RBD residues contacted by 2-3-Fc as compared with BA.1, BA.2, and BA.5. The exceptions are Omicron BA.2.75, XBB and BA.2.3.20. All the three variants carry a novel N460K mutation that sits in the footprint of aRBD-2, and XBB encodes an additional L368I mutation at the edge of aSA3 footprint. Nonetheless, structurally guided Y29G substitution in aRBD-2 saves the binding loss caused by N460K mutation (Fig. 6c), while L368I mutation could not disrupt the binding of aSA3 as the binding is mediated by the main chain carbonyl of L368 (the counterpart residue L355 of SARS1 RBD) (Fig. 1i). These analyses predict that 2(Y29G)-3-Fc could maintain potent neutralization across currently known and reported major variants.

Encouraged by the potent neutralizing activity, we evaluated the protection efficacy of aSA3-Fc and 2-3-Fc in vivo and found that i.p. administered aSA3-Fc or 2-3-Fc at a single dose of 10 mg/kg conferred effective prophylactic and therapeutic protection against Omicron BA.1 infection in hamsters (Fig. 4), thus confirming their protection efficacy preclinically. Nevertheless, we have only tested one dose (10 mg/kg), and the lowest effective doses need to be determined in the future. As 2-3-Fc neutralizes BA1, BA2, and BA5 with comparable activities (Fig. 3f), 2-3-Fc will also provide substantial in vivo protection against BA.2 and BA.5. The IC_50_ of 2-3-Fc in neutralizing BA.2.75 pseudovirus is 1.27 nM (Fig. 6e), which is comparable to that of aSA3-Fc in neutralizing BA.1 (Fig. 2k), so 2-3-Fc is expected to provide substantial protection against BA.2.75 as well, let alone 2(Y29G)-3-Fc that is 4-fold more potent than 2-3-Fc (Fig. 6e).

Antibodies administered through systemic route often result in low concentration in the lungs, so high doses are generally required to achieve effective lung concentrations^49^. Since the primary target of SARS2 is the respiratory tract, direct delivery of antibodies to the respiratory tract may be more effective in preventing SARS2 infection than systemic administration. Here, we demonstrated the high preventive efficacy of 2-3-Fc against Omicron BA.1 through direct i.n. administration. Even at three low doses of 0.5 mg/kg, i.n. delivered 2-3-Fc completely eradicated infectious particles in the trachea and lungs of the infected hamsters (Fig. 5). Further studies are necessary to determine the lowest effective i.n. dose of 2-3-Fc. We did not examine the bio-distribution of 2-3-Fc in i.n. treated hamsters, but it has been reported that macromolecular IgM can be delivered nasally to the upper and lower airways of mice^18^. Nevertheless, due to the different airway anatomical structures between humans and small rodents, the efficacy of 2-3-Fc nasal drops in preventing Omicron infection in humans needs to be further verified, at least in macaques. An alternative to nasal drops is aerosolization. Antibodies in aerosols are theoretically easier to spread throughout the respiratory tract than in liquid. Inhalation of conventional antibodies as well as Nbs has been shown to reduce upper and lower respiratory tract viral loads and lung injury from SARS2 infection^47,50,51^, the protective efficacy of 2-3-Fc inhalation thus is worthy of future investigation.

In summary, we identified a novel robust SARS1/2 bi-neutralizing Nb, aSA3, and further engineered a novel bispecific Nb dimer, 2-3-Fc, which exhibited a broad-spectrum neutralizing potency against all SARS2 VOCs through BA.5. Its derivative 2(Y29G)-3-Fc also potently neutralizes BA.2.75 variant containing N460K mutation, achieving full coverage of all Omicron variants. Moreover, 2-3-Fc can achieve excellent in vivo anti-Omicron efficacy not only by systemic administration but also by nasal delivery. The nasal deliverable 2-3-Fc is a hopeful arsenal complement to vaccines for Omicron prevention to benefit people who are at high risk of transmitting or causing disease spread in the community, such as elderly, immunocompromised individuals, and front-line health-care staff.

## Materials and methods

### Protein preparation

The recombinant proteins of SARS1 RBD (NC_004718, aa 309–540), WT SARS2 RBD (MN908947, aa 321–591), SARS1 RBD-tr2 (tandem repeat aa 306–523), and SARS2 RBD-tr2 (tandem repeat aa 319–537) were prepared as described in our previous study^25^. Briefly, the coding sequences were cloned into the pTT5 vector containing a TEV cleavage site and a human IgG1 Fc at the C-terminus. The recombinant vector was transiently transfected into HEK293F cells with polyethyleneimine (Polyscience). Three days after expression, fusion proteins were purified from cell supernatants using protein A columns (GE Healthcare). After digestion with TEV protease, Fc fragments were removed by a second protein A column purification, and TEV protease was removed by a nickel column (GE Healthcare). Mutations in the RBD of Alpha, Beta, Gamma, Kappa, Lambda, Delta, and Delta plus variants were introduced by PCR. Omicron BA.1, BA.2, and BA.5 RBD proteins were purchased from Sino Biological. The coding sequence of the human ACE2 extracellular domain (NM_001371415, aa 19–615) was also cloned into the pTT5 vector and expressed as an ACE2-tev-Fc fusion (termed ACE2-Fc) and then purified from the cell supernatant using a protein A column. aSA3-Fc, 2-3-Fc, and 2(Y29G)-3-Fc were prepared in the same way without the TEV cleavage site. The linker between aSA3 and Fc is three repeats of “GGGGS”, and the linker between aRBD-2 and aSA3 is four repeats of “GGGGS”. aSA3 with a 6× His-tag used for crystallization was expressed with the pET22b vector in *Escherichia coli* BL21 and purified from bacterial lysate using a nickel column. All recombinant vectors were constructed based on the Gibson Assembly method^52^.

### Phage display

Immuno MaxiSorb plates (Nunc) were coated with 0.1 mL of SARS1 RBD solution (100 μg/mL and 10 μg/mL in the 1st and 2nd round, respectively). Control wells without antigen coating were used in parallel in every round of panning. After blocking with MPBS (PBS supplemented with 5% milk powder) for 2 h at room temperature (RT), 1 × 10^11^ PFU of library phages displaying VHH derived from the PBMCs of alpacas immunized with SARS2 RBD we constructed before^25^ were added for the 1st round of selection. The wells were washed with PBST (PBS supplemented with 0.1% Tween 20) 30 times to remove the unbound phages. Bound phages were eluted by digestion with 100 μL of 0.5 mg/mL trypsin for 1 h at RT. The eluted phages were used to infect *Escherichia coli* TG1 for titer determination and amplification. The 2nd round of panning was performed similarly except that the amount of input phage was 1 × 10^9^ PFU.

Two hundred and sixty individual clones from the 2nd round of panning were picked and identified using monoclonal phage ELISA. The monoclonal phage was rescued with helper phage KM13 and added to the well coated with 2 μg/mL RBD. After 1 h of incubation at RT, the wells were washed four times with PBST, and HRP conjugated anti-M13 antibody (Sino Biological) was added. After washing four times with PBST, TMB (Beyotime) was added to each well and incubated in the dark at RT for 5 min. The chromogenic reaction was stopped with 50 μL of 1 M sulfuric acid, and the OD_450_ was determined by a Synergy H1 plate reader (Biotek). VHHs of positive clones were sequenced and compared.

### ELISA

To test the binding activity of aSA3-Fc to SARS1 RBD or SARS2 RBD, immuno MaxiSorb plates (Nunc) were coated with different RBDs at a final concentration of 2 μg/mL for 4 h at 4 °C. The plates were washed with PBS and then blocked with MPBS for 2 h at RT. aSA3-Fc solutions that were serially diluted 1:4 were added to the plates and incubated for 1 h at RT. After washing with PBST 4 times, the bound aSA3-Fc was detected with a monoclonal HRP-conjugated anti-human IgG1 Fc antibody (Sino Biological). For the ACE2-RBD blocking assay, immuno MaxiSorb plates were coated with SARS1 RBD-tr2 or SARS2 RBD-tr2. The aSA3 solution was serially diluted 1:4 with 20 nM ACE2-Fc solution and then added to the RBD-coated wells and incubated for 1 h. After washing 4 times with PBST, bound ACE2-Fc was detected with HRP conjugated anti-human IgG1 Fc antibody (Sino Biological). After incubation for 1 h at RT, the plates were washed, 100 μL per well of TMB was added and incubated in the dark for 5 min, and 50 μL per well of H_2_SO_4_ (1 M) was added to stop the reaction. The OD_450_ was read by a Synergy H1 plate reader (Biotek). The data were analyzed using GraphPad Prism software.

### SPR

SPR measurements were performed at 25 °C using a BIAcore 8 K system (Cytiva). RBD was diluted to a concentration of 5 μg/ml with sodium acetate (pH 4.5) and immobilized on an activated CM5 chip (Cytiva). All proteins were exchanged into running buffer (PBS containing 0.05% Tween 20, pH 7.4) at a flow rate of 30 μL/min. The blank channel of the chip was used as the negative control. For affinity measurements, a series of dilutions of antibodies were flowed over the sensor chip. After each cycle, the chip was regenerated with 50 mM NaOH buffer for 60 s to 120 s. The sensorgrams were fitted with a 1:1 binding model using BIAcore evaluation software. To determine the competition between aSA3 and aRBD-2 in binding to RBD, aSA3 was injected at a concentration of 200 nM for 120 s to achieve binding saturation, followed by a 1:1 mixture of aSA3 and aRBD-2 at a concentration of 200 nM for 120 s. A rise in signal means there is no competition between the two Nbs.

### Crystallization and data collection

Purified SARS1 RBD-tr2 was mixed with aSA3 in a molar ratio of 1:1.2 to form a complex. To remove excessive aSA3, the mixture was further purified by gel filtration. The protein complex was concentrated to 20 mg/mL for crystallization screening. The sitting-drop vapor diffusion method was applied to obtain the crystals of complexes by mixing 0.2 µL of protein complexes with an equal volume of reservoir solution. Crystals were achieved in a condition composed of 0.2 M Li acetate, cacodylate (pH 6.5), 20% PEG6000 for ~1 month at 18 °C. For data collection, single crystals were flashed-cooled in liquid nitrogen after immersion in cryoprotectant composed of 15% (v/v) glycerol in the reservoir solution for a few seconds. Diffraction data were collected at BL02U1 beamline at the wavelength of 0.97911 Å at the Shanghai Synchrotron Radiation Facility (SSRF).

### Structural determination

Data were processed with XDS^53^. Initial phases were solved by the molecular replacement method with Phaser^54^ from the CCP4i program package^55^ using SARS1 RBD (PDB ID: 7LM9) and Nb Re5D06 (PDB ID: 7OLZ) as search models for the SARS1 RBD-tr2:aSA3 complex. Subsequent model building and refinement were achieved using COOT and Phenix^56^. The structural data of RBD-tr2:aSA3 complexes have been deposited in PDB under accession codes 7X4I. All structural figures were prepared with PyMOL.

### Pseudovirus neutralization assay

Pseudoviruses were used to evaluate the neutralizing activities of our antibodies against SARS1, Omicron BA.1, BA.2, BA.5, and BA.2.75. HIV-1-based pseudoviruses carrying SARS1, BA.1 or BA.2 spike and luciferase reporter genes were prepared as we previously described^57^, while HIV-1-pseudotyped with BA.5 and BA.2.75 spike were purchased from Vazyme Jiangsu, China. Pseudovirus neutralization assays were performed as we previously described^57^. Briefly, ACE2-293T cells were cultured overnight in 96-well plates at 2.5 × 10^4^ per well. The antibodies serially diluted threefold with DMEM plus 10% FBS were incubated with an equal volume of pseudovirus (SARS1, BA.1, BA.2, BA.5, and BA.2.75 pseudovirus sufficient to generate 1,300,000–2,000,000, 250,000–400,000, 300,000–400,000, 4,600,000–5,000,000, and 1,000,000–1,500,000 relative light units, respectively) at 37 °C for 1 h. The antibody-pseudovirus mixtures were then added to the ACE2-293T cell monolayer. After 2 d of culture, the cells were lysed and treated using Bright-Lite detection reagent (Vazyme, DD1204). Luciferase activity was measured by a microplate luminescence detector (TECAN, SPARK 10 M). Cells without viruses and antibodies were used as blank controls, and cells without antibodies were used as virus controls. The neutralization percentage was calculated by the formula: neutralization (%) = [1 – (sample RLU−blank RLU)/(positive control RLU−blank RLU)] (%).

### PRNT

PRNT was employed to test the neutralizing activities of our antibodies against authentic WT SARS2, Beta, Delta and Omicron BA.1 variants as described in a previous study^58^ with slight modification. Briefly, Vero E6 cells were cultured overnight in 24-well plates at 1.5 × 10^5^ per well. Antibodies serially diluted 1:5 in DMEM containing 2.5% FBS were incubated with equal volumes of 75 PFU of SARS2 WT virus (IVCAS 6.7512), Beta virus (NPRC2.062100001), Delta virus (GWHBEBW01000000), and Omicron BA.1 virus (CCPM-B-V-049-2112-18) at 37 °C for 1 h. Then, the mixture was added to the wells. Cells infected with virus without antibody addition were used as controls. After an additional 1 h incubation at 37 °C, the antibody-virus mixture was removed, and DMEM containing 2.5% FBS and 0.9% carboxymethyl cellulose was added. Plates were fixed with 8% paraformaldehyde, stained with 0.5% crystal violet and rinsed thoroughly with water 3 days later. Plaques were then enumerated, and the neutralization IC_50_ was calculated using GraphPad Prism software. The inhibition percentage was calculated by the formula: neutralization (%) = (1−sample plaque/blank plaque) (%).

### Micro-neutralization test

To evaluate the neutralizing activities of our antibodies against WIV1 (a bat coronavirus closely related to SARS1), a micro-neutralization test was conducted as described in a previous study with some modifications^59^. Briefly, Vero E6 cells were cultured overnight in 96-well plates seeded at 1.8 × 10^5^ per well. Antibodies serially diluted 1:3 in DMEM were incubated with 100 TCID_50_ of authentic WIV1 at 37 °C for 2 h. Then, the antibody-virus mixture was added to a 96-well microtiter plate containing an equal volume of confluent Vero E6 cells with 6 repeats and incubated at 37 °C in a CO_2_ incubator for 4 d. After 4 d of culture, the cytopathic effect of each well was recorded under a microscope by two independent observers. The titer was calculated as the highest dilution that eliminated the cytopathic effect in 50% of the wells (IC_50_) by the Reed and Muench method.

### Hamster studies

To test aSA3-Fc and 2-3-Fc for protection in vivo via i.p. administration, female Syrian golden hamsters (5 to 6 weeks old) were anesthetized with 3%–5% isoflurane and infected i.n. with 1 × 10^4^ PFU of SARS2 Omicron BA.1 virus. A 10 mg/kg dose of aSA3-Fc or 2-3-Fc was i.p. administered to the hamsters at 24 h pre-infection (prophylactic group) or 3 h post-infection (therapeutic group), respectively. Animals were weighed daily and euthanized with isoflurane overdose at 4 dpi, and tissues (trachea and lungs) were harvested and homogenized in 1 mL PBS. The supernatants were collected to measure viral RNA copies and infectious virus titers.

To test 2-3-Fc for protection in vivo via i.n. delivery, four dose levels (5 mg/kg, 2 mg/kg, 1 mg/kg, 0.5 mg/kg) of 2-3-Fc in 100 μL PBS were i.n. administered to female hamsters (5 to 6 weeks old). Three hours later, the hamsters were anesthetized with isoflurane and i.n. inoculated with 1 × 10^4^ PFU of SARS2 Omicron BA.1 virus. Then, two additional nasal administrations were conducted at 24 and 48 h post-infection. Animals were weighed daily and euthanized with isoflurane overdose at 3 dpi, and tissues (trachea and lungs) were harvested and homogenized in 1 mL PBS. The supernatants were collected to measure viral RNA copies and infectious virus titers. All operations were performed in the biosafety level 3 (BSL-3) facility, and the protocols were approved by the Institutional Review Board at Wuhan Institute of Virology (assurance number: WIVAF45202202).

### Virus RNA copies and titers

Viral RNA in the tissue homogenates was quantified by one-step real-time RT‒PCR as described previously^60^. Briefly, viral RNA was purified using the QIAamp Viral RNA Mini Kit (Qiagen) and quantified with the HiScript® II One Step qRT‒PCR SYBR® Green Kit (Vazyme Biotech Co., Ltd) with the primers ORF1ab-F (5′-CCCTGTGGGTTTTACACTTAA-3′) and ORF1ab-R (5′-ACGATTGTGCATCAGCTGA-3′). The amplification procedure was set up as follows: 50 °C for 3 min, 95 °C for 30 s followed by 40 cycles consisting of 95 °C for 10 s and 60 °C for 30 s.

The virus titer was determined with a plaque assay as previously described with slight modification^44^. Briefly, virus samples were serially ten-fold diluted with DMEM containing 2.5% FBS and inoculated into Vero E6 cells cultured overnight at 1.5 × 10^5^ per well in 24-well plates; after incubating at 37 °C for 1 h, the inoculate was replaced with DMEM containing 2.5% FBS and 0.9% carboxymethyl-cellulose. The plates were fixed with 8% paraformaldehyde and stained with 0.5% crystal violet 3 d later. The virus titer was calculated with a dilution gradient of 10–100 plaques.

### Statistical analysis

All statistical analyses were performed in GraphPad Prism. An unpaired *t*-test with Welch’s correction for unequal standard deviations was used for comparisons of two groups. The asterisks shown in the figures refer to the level of significance: *P ≤ 0.05; **P ≤ 0.01; ***P ≤ 0.0001.

## Supplementary information

Supplementary Information
