# Supplementary material for: A bispecific nanobody dimer broadly neutralizes SARS-CoV-1 & 2 variants of concern and offers substantial protection against Omicron via low-dose intranasal administration
Source: Cell Discov. 2022 Dec 9;8:132. doi: 10.1038/s41421-022-00497-w (PMC9734137; doi:10.1038/s41421-022-00497-w)
Supplement: Supplementary file 1 — Supplementary Information [file 41421_2022_497_MOESM1_ESM.pdf]

Supplementary Information for

A bispecific nanobody dimer broadly neutralizes SARS-CoV-1 & 2 variants of concern and offers substantial protection against Omicron via low-dose intranasal administration

Huan Ma[1#], Xinghai Zhang[2#], Weihong Zeng[3#], Junhui Zhou[2,4#], Xiangyang Chi[5#], Shaohong Chen[2,4], Peiyi Zheng[3], Meihua Wang[3], Yan Wu[2], Dan Zhao[3], Fanwu Gong[3], Haofeng Lin[2,4], Hancong Sun[5], Changming Yu[5], Zhengli Shi[6], Xiaowen Hu[1], Huajun Zhang[2]*, Tengchuan Jin[3,7]*, Sandra Chiu[3]*

[1] Department of Pulmonary and Critical Care Medicine, The First Affiliated Hospital of USTC, Division of Life Sciences and Medicine, University of Science and Technology of China, Hefei, Anhui 230001, China.

[2] State Key Laboratory of Virology, Wuhan Institute of Virology, Center for Biosafety Mega-Science, Chinese Academy of Sciences, Wuhan, 430062, China.

[3] Division of Life Sciences and Medicine, University of Science and Technology of China, Hefei, Anhui 230027, China.

[4] University of Chinese Academy of Sciences, Beijing, 100049, P. R. China.

[5]Institute of Biotechnology, Academy of Military Medical Sciences, Beijing 100071, China

[6] CAS Key Laboratory of Special Pathogens, Wuhan Institute of Virology, Chinese Academy of Sciences, Wuhan, 430062, China.

[7] Institute of Health and Medicine, Hefei Comprehensive National Science Center, Hefei, Anhui, China.

*To whom correspondence should be addressed:

Prof. Sandra Chiu: Division of Life Sciences and Medicine, University of Science and Technology of China, Hefei, 230027, China; Email: qiux@ustc.edu.cn; Tel: +86-551-63603264;

Prof. Tengchuan Jin: Division of Life Sciences and Medicine, University of Science and Technology of China, Hefei, 230027, China; Email: jint@ustc.edu.cn; Tel: +86-551-63600720;

Prof. Huajun Zhang: Wuhan Institute of Virology, Chinese Academy of Sciences, Wuhan, Hubei, China; Email: hjzhang@wh.iov.cn

[#]These authors contributed equally to this work.

Supplementary Table S1. Data collection and refinement statistics

	SARS1-RBD-tr2: aSA3
Data collection	BL02U1
Wavelength (Å)	0.97911
Space group	$P2_12_12_1$
Unit cell parameters	
a, b, c (Å)	101.70, 112.01, 151.04
α, β, γ (°)	90, 90, 90
Resolution range (Å)	151.04-3.38 (3.56-3.38)
Monomers in an asymmetric unit	4
Unique reflections	24667 (3528)
Average redundancy	7.0 (7.5)
Completeness (%)	99.5 (99.4)
R_{merge} (%)[a]	24.4 (133.4)
I/σ (I)	8.7 (2.3)
Wilson B factor (Å^2)	85.1
Refinement Statistics	
Resolution range (Å)	89.97-3.38
R_{factor} (%)[b]	26.70
R_{free} (%)[c]	32.09
RMSD bond lengths (Å)	0.005
RMSD bond angles (°)	0.731
Mean B factors (Å^2)	
Protein	100.01
Ligand	0
Water	0
No. of non-hydrogen protein atoms	9779
No. of ligand atoms	0
No. of water oxygen atoms	0
Ramachandran plot[d]	
Favored (%)	95.1
Outliers (%)	0
PDB entry	7X4I

[a]$R_{\mathrm{merge}} = \sum_{hkl}\sum_i |I_i(hkl) - \langle I(hkl)\rangle| / \sum_{hkl}\sum_i I_i(hkl)$, where $I_i(hkl)$ is the intensity of the *ith* observation and <I(hkl)> is the mean value for reflection hkl.

[b]$R_{\mathrm{work}} = \sum_{hkl}\big||F_{obs}| - |F_{cacl}|\big| / \sum_{hkl}|F_{obs}|$, where F_{obs} and F_{calc} are the observed and calculated structure-factor amplitudes, respectively.

[c]R_{free} is calculated in the same way as R_{work} with 5% reflections, which were selected randomly from the refinement process.

[d]The categories were defined by PROCHECK.

The numbers in brackets are the parameters for the “Resolution range,” “Unique reflection,” “Average redundancy,” “Completeness,” “R_{merge},” and “I/σ(I)” of the highest resolution shell.

Supplementary Table S2. Interaction bonds formed between aSA3 and SARS1 RBD.

SARS-CoV-1 RBD		aSA3		Bonds
Residue	atom	Residue	atom	
Leu355	O	Arg103	NH2	Hydrogen bond
Tyr356	O	Arg103	NE	Hydrogen bond
Tyr356	OH	Arg103	N	Hydrogen bond
Tyr356	OH	Asp52	OD2	Hydrogen bond
Ser358	O	Arg103	NH2	Hydrogen bond
Phe361	O	Arg103	NH1	Hydrogen bond
Ser362	OG	Asn106	ND2	Hydrogen bond
Ser362	O	Leu105	N	Hydrogen bond
Thr363	OG1	Asn106	ND2	Hydrogen bond
Thr363	OG1	Ser104	OG	Hydrogen bond
Phe364	O	Arg103	N	Hydrogen bond
Phe364	N	Arg103	O	Hydrogen bond
Lys365	NZ	Asp109	OD1	Hydrogen bond
Cys366	O	Tyr101	O	Hydrogen bond
Arg395	NE	Phe108	O	Hydrogen bond
Arg395	NH2	Tyr110	N	Hydrogen bond
Arg395	NH1	Asp111	OD2	Salt bridge
Ile489	N	Glu44	OE2	Hydrogen bond
Gly490	N	Glu44	OE2	Hydrogen bond
Tyr494	OH	Asn106	OD1	Hydrogen bond
Val394/Tyr494		Phe108		hydrophobic interaction
